# PCSK9i promoting the transformation of AS plaques into a stable plaque by targeting the miR-186-5p/Wipf2 and miR-375-3p/Pdk1/Yap1 in ApoE−/− mice

**DOI:** 10.3389/fmed.2024.1284199

**Published:** 2024-02-26

**Authors:** Yanlong Zhao, Ning Liu, Jifeng Zhang, Lei Zhao

**Affiliations:** ^1^Department of Cardiology, The Second Hospital of Jilin University, Changchun, Jilin, China; ^2^School of Pharmaceutical Sciences, Jilin University, Changchun, Jilin, China

**Keywords:** atherosclerosis, PCSK9i, miRNA, atorvastatin, atherosclerotic plaque

## Abstract

**Background:**

Atherosclerosis (AS) is a multifaceted disease characterized by disruptions in lipid metabolism, vascular inflammation, and the involvement of diverse cellular constituents. Recent investigations have progressively underscored the role of microRNA (miR) dysregulation in cardiovascular diseases, notably AS. Proprotein convertase subtilisin/kexin type 9 inhibitors (PCSK9i) can effectively reduce circulating levels of low-density lipoprotein cholesterol (LDL-C) and lipoprotein (a) [Lp (a)], potentially fostering a more enduring phenotype for AS plaques. However, the underlying mechanisms by which PCSK9i enhances plaque stability remain unclear. In this study, we used microarray and bioinformatics techniques to analyze the regulatory impacts on gene expression pertinent to AS, thereby unveiling potential mechanisms underlying the plaque-stabilizing attributes of PCSK9i.

**Methods:**

ApoE−/− mice were randomly allocated into control, AS, PCSK9i, and Atorvastatin groups. The AS model was induced through a high-fat diet (HFD), succeeded by interventions: the PCSK9i group was subjected to subcutaneous SBC-115076 injections (8 mg/kg, twice weekly), and the Atorvastatin group received daily oral Atorvastatin (10 mg/kg) while on the HFD. Subsequent to the intervention phase, serum analysis, histological assessment using hematoxylin and eosin (H&E) and Oil Red O staining, microarray-centered miRNA analysis utilizing predictions from TargetScan and miRTarBase, and analyses using Gene Ontology (GO) and Kyoto Encyclopedia of Genes and Genomes (KEGG) were executed to illuminate potential pathways. Real-time fluorescence quantitative PCR (RT-qPCR) was employed to quantify the expression levels of target genes.

**Results:**

In comparison to the control group, the AS group displayed a significant elevation in blood lipid levels. Both PCSK9i and Atorvastatin effectively attenuated blood lipid levels, with PCSK9i exhibiting a more pronounced lipid-lowering impact, particularly concerning TG and LDL-C levels. Over the course of AS progression, the expression levels of mmu-miR-134, mmu-miR-141-5p, mmu-miR-17-3p, mmu-miR-195-3p, mmu-miR-210, mmu-miR-33–5p, mmu-miR-410, mmu-miR-411-5p, mmu-miR-499, mmu-miR-672-5p, mmu-miR-675-3p, and mmu-miR-301b underwent dynamic fluctuations. PCSK9i significantly down-regulated the expression of mmu-miR-186-5p, mmu-miR-222, mmu-miR-375-3p, and mmu-miR-494-3p. Further enrichment analysis disclosed that mmu-miR-186-5p, mmu-miR-222, mmu-miR-375-3p, and mmu-miR-494-3p were functionally enriched for cardiovascular smooth muscle cell proliferation, migration, and regulation. RT-qPCR results manifested that, in comparison to the AS group, PCSK9i significantly upregulated the expression of Wipf2, Pdk1, and Yap1 (*p* < 0.05).

**Conclusion:**

Aberrant miRNA expression may play a pivotal role in AS progression in murine models of AS. The subcutaneous administration of PCSK9i exerted anti-atherosclerotic effects by targeting the miR-186-5p/Wipf2 and miR-375-3p/Pdk1/Yap1 axes, thereby promoting the transition of AS plaques into a more stable form.

## 1 Introduction

Atherosclerosis (AS) represents a multifaceted lipid-related inflammatory ailment accountable for a spectrum of grave conditions, including myocardial infarction and stroke. Annually, it imposes substantial economic burdens upon global populations (1, 2). AS is closely associated with perturbations in lipid metabolism, inflammatory responses, non-genetic predisposing factors (e.g., smoking, high-fat diet, and environmental determinants), and epigenetic modulation (3–5). Epigenetic processes play an indispensable role in the pathophysiological cascade of AS.

Proprotein convertase subtilisin/kexin type 9 inhibitors (PCSK9i), a novel lipid-lowering class, significantly reduce LDL-C levels in familial hypercholesterolemia (FH) or atherosclerosis (AS) patients (6). By targeting AS-related genes, PCSK9i enhances LDL receptor (LDLR) function for efficient LDL-C clearance (7). Fourier (8) and Odyssey (9) trials reveal decreased LDL-C levels and reduced major adverse cardiovascular events (MACE) with PCSK9i treatment. Intravascular ultrasound (IVUS) and optical coherence tomography (OCT) studies show that PCSK9i improves coronary AS plaque stability by increasing fibrous cap thickness and lumen area while decreasing lipid arc (10, 11). However, precise molecular mechanisms underlying this phenomenon require further investigation.

MicroRNAs (miRNAs or miRs) constitute a class of non-coding RNAs pivotal to post-transcriptional regulation and disease pathogenesis. Their functions encompass the regulation of cell growth, proliferation, differentiation, migration, senescence, apoptosis, and angiogenesis (12). Recent investigations have progressively unveiled substantial involvement of miRNA dysregulation in cardiovascular diseases, with particular emphasis on AS (3, 13). This has provided new molecular visions about atherosclerosis and has been presented as a novel therapeutic approach.

In recent time, the widespread embrace of microarray technology has furnished a high-throughput tool in molecular biology, capacitated to simultaneously gage the expression profiles of numerous genes (14). Through harnessing this microarray technology, we are endowed with the expeditious and precise means to scrutinize the impact of PCSK9i on AS, thereby amplifying our comprehension of the disorder and pinpointing innovative therapeutic avenues. The primary objective of this study was to deploy microarray and bioinformatics methodologies to analyze the regulatory effects of PCSK9i on pertinent genes implicated in AS, thereby elucidating the potential mechanisms underpinning the promotion of plaque stability by PCSK9i. These revelations furnish a conceptual foundation for the formulation of forthcoming treatment strategies.

## 2 Materials and methods

### 2.1 Reagents

SBC-115076 was acquired from Selleck Chemicals (Houston, TX, United States). Atorvastatin calcium (Lipitor) was procured from Pfizer. The cholesterol Kit was obtained from Nanjing Jiancheng Bioengineering Institute (Nanjing, China). Mouse targets for the atherosclerosis-related gene qPCR array were sourced from Wcgene Biotech (Shanghai, China). The PCR primers were provided by the Beijing Genomics Institute (Beijing, China). All other chemicals were of analytical grade and were purchased from standard commercial suppliers.

### 2.2 Animals and experimental procedures

To mitigate the potential influence of estrogen and comprehensively explore the expression of miRNAs at different stages of AS *in vivo*, male ApoE−/− mice, 8 weeks of age, with a body weight of 20.73 ± 0.35 g, were obtained from Beijing Vital River Laboratory Animal Technology Co., Ltd. (Beijing, China). After a 2-week acclimation period, the mice were randomly assigned to four groups (*n* = 7) using a random number table: control, AS, PCSK9i, and Atorvastatin. All groups were subjected to the same Western high-fat diet for 12 weeks to induce atherosclerosis development, except the control group. Following the 12-week feeding period, euthanasia was performed for the AS group, while the PCSK9i and Atorvastatin groups continued to receive their respective interventions along with the high-fat diet. The PCSK9i group received subcutaneous injections of the PCSK9 inhibitor (SBC-115076) at a dosage of 8 mg/kg twice weekly (calculated as 9.1 times the clinical dosage, with units in g/kg/d). The Atorvastatin group received a daily oral dose of 10 mg/kg of atorvastatin. After 8 weeks of intervention, the PCSK9i and Atorvastatin group mice were euthanized randomly. Cardiac and aortic tissues were swiftly collected and flash-frozen in liquid nitrogen for subsequent analysis. All animal procedures were granted ethical approval by the Ethics Committee of the School of Basic Medical Sciences, Jilin University, and adhered to the ethical requisites for animal protection and use.

### 2.3 Detection of plasma levels of cholesterol

At the intervention’s conclusion, all mice underwent an overnight fast with water deprivation, followed by inhalation of a suitable amount of isoflurane anesthesia. After anesthesia, blood samples were drawn from the orbital vein and promptly centrifuged. Plasma levels of total cholesterol (TC), triglycerides (TG), LDL-C, and high-density lipoprotein cholesterol (HDL-C) were quantified using a Cholesterol Kit (Nanjing Jiancheng Bioengineering Institute, Nanjing City, China).

### 2.4 He staining and oil red O staining

The aortic roots of mice were fixed in 4% paraformaldehyde, subjected to gradual alcohol dehydration and paraffin embedding. Precise 8 μm-thick sequential sections were prepared, followed by immersion in hematoxylin and eosin solution, and subsequent mounting with a neutral mounting medium for microscopy observation. Oil Red O staining was performed on fixed sections, which were rinsed with pH 7.4 PBS and air-dried. Subsequently, they were stained with a 0.3% Oil Red O solution for 3 min. After washing and mounting with 87% glycerol, stained sections were microscopically examined. The percentage of the stained lesion area relative to the total area was measured using ImageJ software, and the data are presented as mean ± SEM.

### 2.5 Isolation of total RNA and miRNA expression profiling

Mouse aortic tissues, dissected from the region spanning from the ascending aorta to the first bifurcation of the descending aorta, were retrieved from liquid nitrogen, homogenized after thawing, and total RNA was extracted using TRIzol^®^ Reagent (Invitrogen, CA, United States). The aortic valve was deliberately excluded from the dissected tissues during the extraction process. Gene expression profiles were analyzed using a microarray (Wcgene Biotech, Shanghai, China) following the manufacturer’s instructions. Target gene relative expression levels and fold change (FC) were determined using the 2^ΔΔCt^ method. Student’s t-test calculated *p*-values for the target genes. Target genes with an FC >1.2 or < 0.833, and *p* < 0.05 were designated as differentially expressed miRNAs (DEMs).

### 2.6 Prediction of target genes for DEMs and enrichment analysis

DEM target genes were predicted using TargetScan 8.0^1^ and miRTarBase,^2^ with interactions validated by pertinent literature. Predicted regulatory networks were visualized using Cytoscape 3.9.1 for a comprehensive understanding of potential regulatory mechanisms. For functional annotation and pathway enrichment analyses, DAVID 6.8^3^ was employed, using Gene Ontology (GO) and Kyoto Encyclopedia of Genes and Genomes (KEGG) databases. Enriched results were visualized using RStudio software.

### 2.7 Real-time fluorescence quantitative PCR (RT-qPCR)

Total RNA quality and concentration were assessed using spectrophotometry and gel electrophoresis. Subsequently, cDNA synthesis was performed using a SPARKscript II RT Kit (Spark Jade, China) as per the manufacturer’s protocol. Real-time quantitative PCR (RT-qPCR) was conducted on an iQ5 Multicolor RT-PCR Detection System (Bio-Rad, Hercules, CA, United States) using a SYBR Green qPCR Mix kit (Sparkjade, China). Primer sequences for target genes were sourced from PrimerBank,^4^ with preference for validated sequences (Table 1). Raw threshold cycle (Ct) values were subjected to comparative quantification, with target gene expression levels normalized to the internal reference gene Gapdh using the 2^–ΔΔCt^ method.

**Table 1:** Reverse transcription quantitative polymerase chain reaction (RT-qPCR).

Name	Sequence 5′ -3′
Gapdh	CTCGCTTCGGCAGCACA
	AACGCTTCACGAATTTGCGT
Wipf2 Pdk1 Yap1	GAGAAGGACATCCTGCTCCA GTGTCTCTGTGGCAGCTCA GGACTTCGGGTCAGTGAATGC TCCTGAGAAGATTGTCGGGGA ACCCTCGTTTTGCCATGAAC TGTGCTGGGATTGATATTCCGTA

### 2.8 Statistical analysis

Data are presented as mean ± standard error of the mean (SEM). Intergroup differences were analyzed using Student’s t-test for pairwise comparisons and one-way analysis of variance (ANOVA) for multiple group comparisons. A significance level of *p* < 0.05 was considered statistically significant.

## 3 Results

### 3.1 PCSK9i reduced serum lipid levels and ameliorated atherosclerotic lesions in ApoE −/−mice

**Figure 1:**
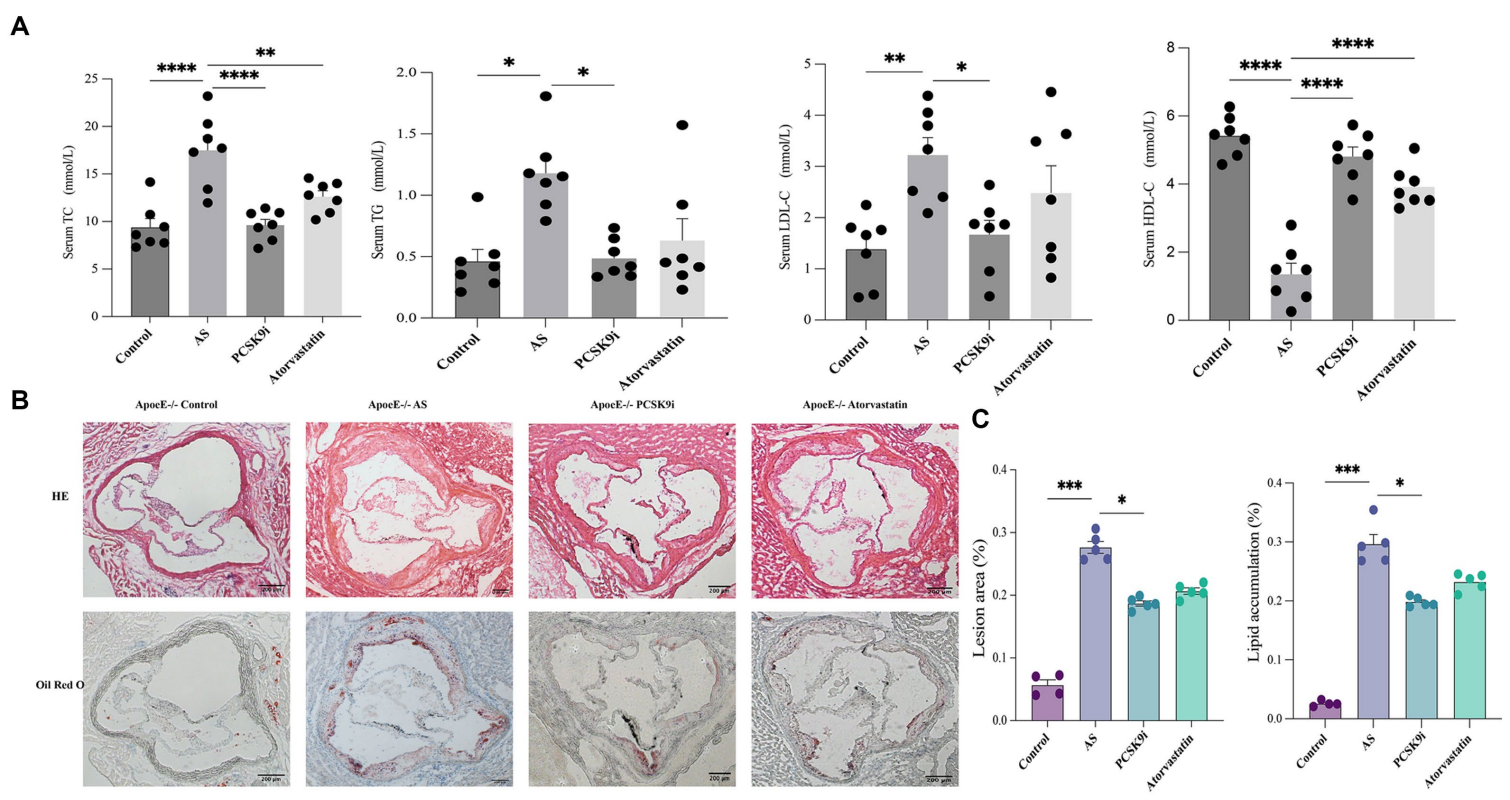
PCSK9i alleviated atherosclerotic lesions in ApoE−/− mice fed a high-fat diet. **(A)** The concentrations of TC, TG, LDL-C, and HDL-C in the serum of the four groups of mice were detected using a kit. **(B)** Histology of HE and oil red O staining in aortic roots of the four groups of mice. **(C)** Quantification of HE and oil red O staining in aortic roots of the four groups of mice. ApoE−/− mice were fed a high-fat diet to establish an atherosclerosis (AS) mouse model. Data in **A** and **C** were analyzed using one-way ANOVA and Tukey’s *post-hoc* test, *p < 0.05; PCSK9i, Subcutaneous injection of SBC-115076 ApoE−/− mice for 8 weeks; TG, Triglyceride; TC, Total cholesterol; LDL-C, Low-density lipoprotein cholesterol; HDL-C, High-density lipoprotein cholesterol.

Compared to the control group, the AS group had higher levels of TC, TG, and LDL-C in their blood, along with lower levels of HDL-C (*p* < 0.05). In contrast, the atorvastatin group had lower TC levels and higher HDL-C levels, with no significant changes in TG and LDL-C (*p* > 0.05). But the most intriguing results came from the PCSK9i group. They showed a significant reduction in TC, TG, and LDL-C levels compared to the AS group, and their HDL-C levels increased significantly (*p* < 0.05; Figure 1A).

Regarding atherosclerotic lesions, HE and Oil Red O staining revealed a notable increase in the lesion area in the AS group compared to that in the control group (*p* < 0.05). In contrast, both the PCSK9i and Atorvastatin groups exhibited a reduction in lesion area compared to the AS group, with PCSK9i demonstrating a more pronounced reduction that was statistically significant (*p* < 0.05). Notably, the Atorvastatin group showed no significant difference (*p* > 0.05) in lesion area compared to AS (Figures 1B,C). This disparity may be attributed to the duration of the drug intervention.

### 3.2 DEMs analysis

**Figure 2:**
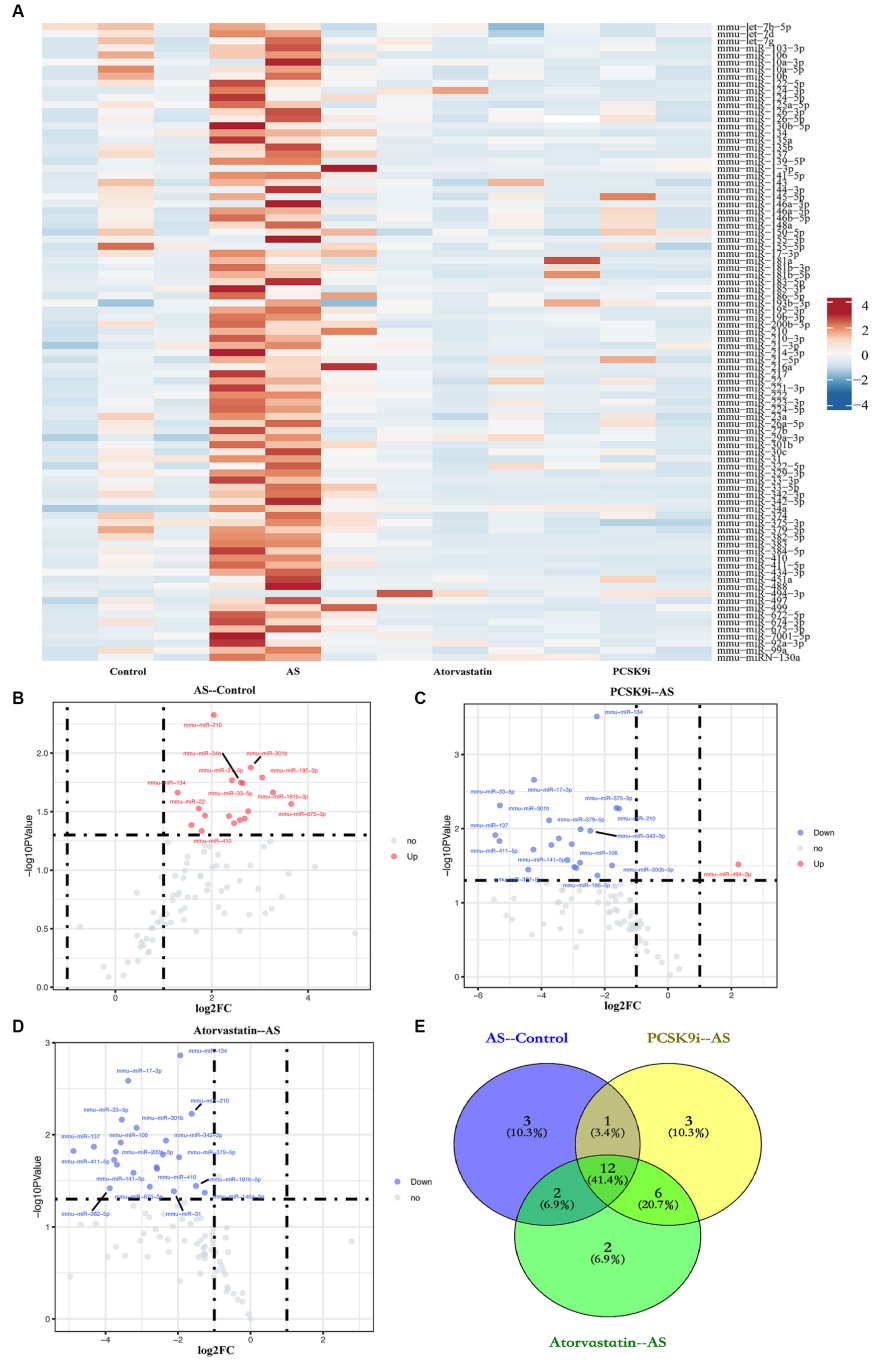
Identification of DEMs. **(A)** Heatmap of miRNA expression in aortic tissues from four groups of ApoE−/− mice. **(B–D)** The volcano plots show the fold change (FC) and statistical significance of DEMs between each group (**B**: AS compared to the control; **C**: PCSK9i compared to the AS; **D**: Atorvastatin compared to the AS). **(E)** Venn diagram showing number of genes overlapped in each group. Spots above the horizontal midline represent statistically significant DEMs. FC >1.2 or < 0.833 and *p* < 0.05 represent the downregulated and upregulated significant DEMs, respectively.

To understand how PCSK9i affects AS lesions, we analyzed the RNA from aortic tissues of ApoE−/− mice in the control, AS, PCSK9i, and Atorvastatin groups using microarray analysis. Our findings showed clear differences in the levels of certain miRNAs among these groups. When compared to the control group, the AS group had 18 miRNAs showing increased levels. On the other hand, the PCSK9i group had one gene with significantly higher levels and 21 genes with significantly lower levels compared to the AS group. In contrast, the Atorvastatin group had 22 genes with lower levels compared to the AS group (Figures 2A–D). Additionally, we noticed changes in 12 miRNAs during AS development and treatment, indicating their importance in how AS develops and progresses (Figure 2E). Particularly, PCSK9i treatment significantly lowered the levels of mmu-miR-186-5p, mmu-miR-222, and mmu-miR-375-3p, while significantly increasing mmu-miR-494-3p. However, the atorvastatin group did not show significant differences in the levels of these miRNAs (Table 2). These distinct responses in miRNA levels in the PCSK9i group compared to the atorvastatin group suggest that these miRNAs might play a role in improving the stability of AS plaques under the influence of PCSK9i.

**Table 2:** Variations in miRNA expression levels across the groups.

miRNA id	AS—Control	PCSK9i—AS	Atorvastatin—AS	Main function in AS
mmu-miR-134*	up	down	down	Regulate lipid accumulation (15)
mmu-miR-141-5p*	up	down	down	Regulate VSMC function (16)
mmu-miR-17-3p*	up	down	down	/
mmu-miR-195-3p*	up	down	down	Regulate macrophage inflammation (17)
mmu-miR-210*	up	down	down	Regulate macrophage mitochondrial function (18)
mmu-miR-33–5p*	up	down	down	Regulate cholesterol efflux (19)
mmu-miR-410*	up	down	down	Regulate endothelial cell proliferation and apoptosis (20)
mmu-miR-411-5p*	up	down	down	/
mmu-miR-499*	up	down	down	Regulate endothelial cell proliferation and migration (21)
mmu-miR-672-5p*	up	down	down	/
mmu-miR-675-3p*	up	down	down	/
mmu-miR-301b*	up	down	down	/
mmu-miR-146a-5p	up	ns	down	Regulate lipid accumulation (22)
mmu-miR-181b-3p	up	ns	down	/
mmu-miR-186-5p***	up	down	ns	Regulate endothelial cell proliferation and apoptosis (23)
mmu-miR-21-5p	up	ns	ns	Regulate inflammatory response (24)
mmu-miR-22	up	ns	ns	Regulate VSMC proliferation and migration (25)
mmu-miR-34a	up	ns	ns	Regulate macrophage cholesterol efflux and inflammation (26)
mmu-miR-106	ns	down	down	/
mmu-miR-137	ns	down	down	Regulate inflammatory response and oxidative stress (27)
mmu-miR-200b-3p	ns	down	down	Regulate lipid accumulation and cholesterol efflux (28)
mmu-miR-222***	ns	down	ns	Regulate VSMC proliferation and migration (29)
mmu-miR-342-3p	ns	down	down	Regulate macrophage inflammation response and lipid uptake (30)
mmu-miR-375-3p***	ns	down	ns	Regulate VSMC phenotypic transformation (31)
mmu-miR-379-5p	ns	down	down	Regulate VSMC proliferation, migration, and invasion (32)
mmu-miR-382-5p	ns	down	down	Regulate lipid accumulation (33)
mmu-miR-494-3p***	ns	up	ns	Regulate proinflammatory macrophage polarization (34)
mmu-miR-31	ns	ns	down	Regulate macrophage apoptosis (35)

*Denotes dynamically modulated miRNAs investigated in the study, while *** represents miRNAs uniquely regulated by PCSK9i.

### 3.3 Identification of target genes and functional enrichment analysis

**Figure 3:**
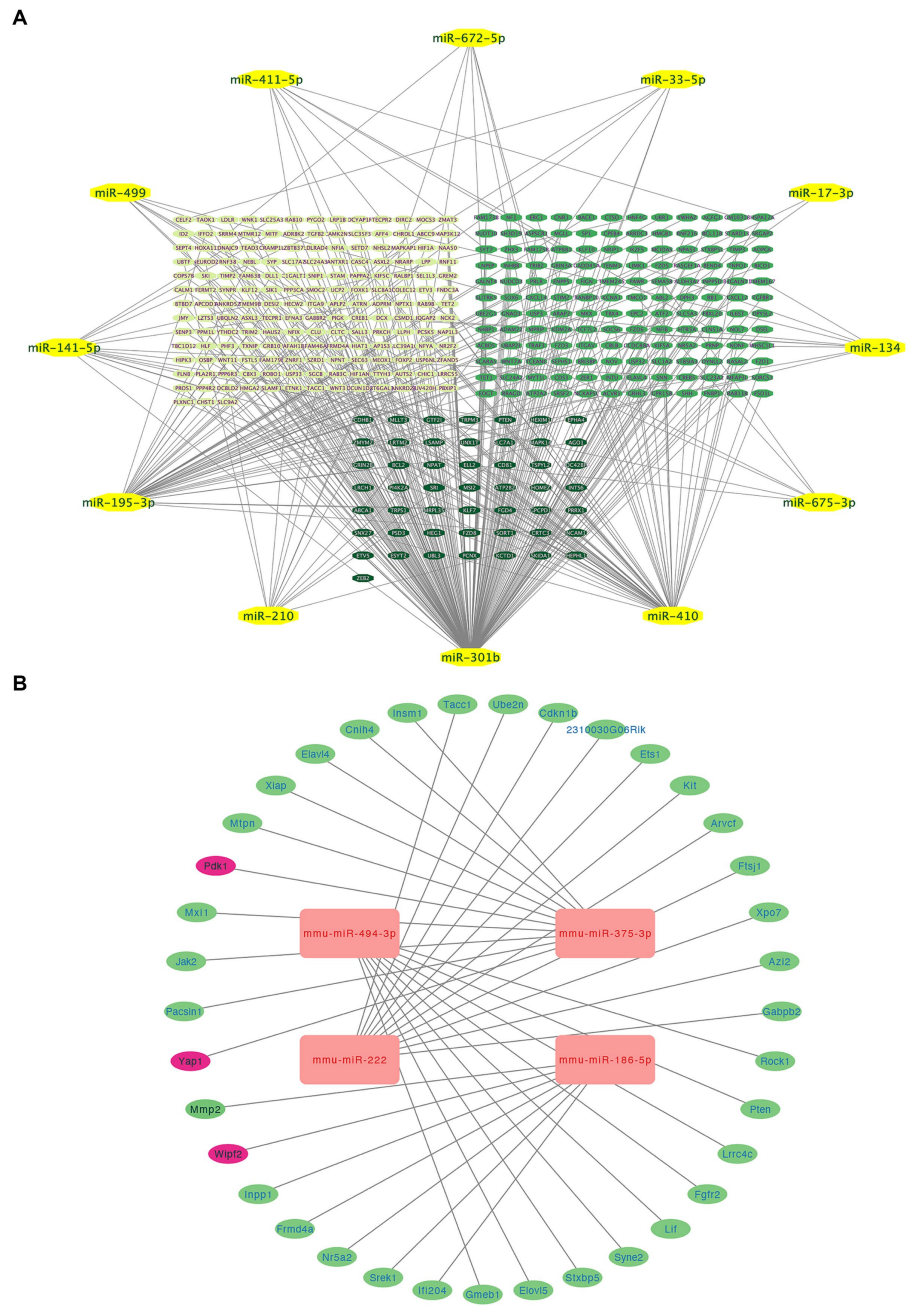
Integrated visualization showcasing miRNA-target gene interactions and enrichment analysis. **(A)** Relationships between various miRNAs and their respective target genes. **(B)** Network visualization of four uniquely regulated miRNAs by PCSK9i and their downstream target genes. MiRNAs are represented in yellow **(A)** and red **(B)**, and the lines indicate regulatory connections between miRNAs and target genes.

**Figure 4:**
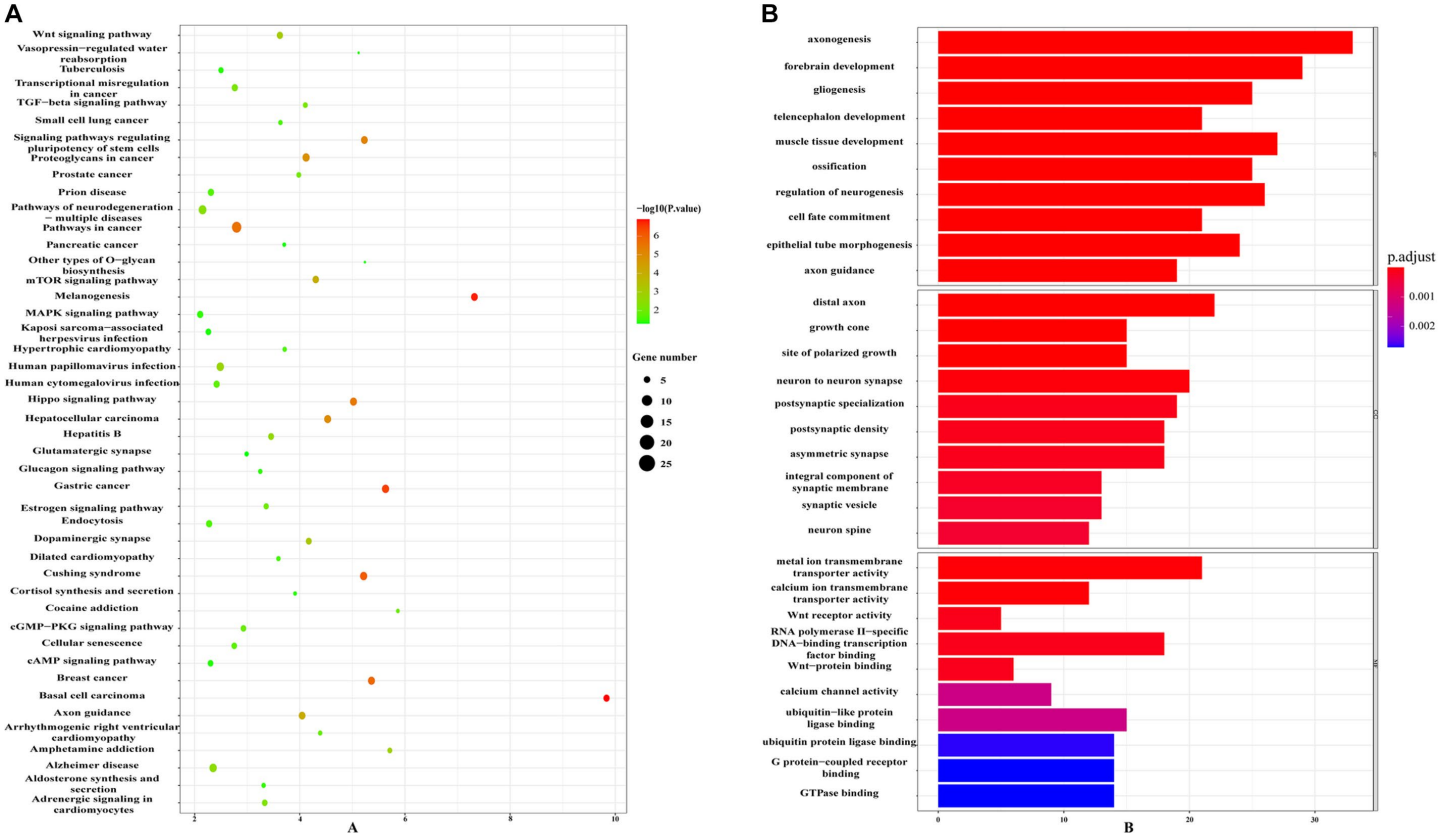
Enrichment analysis of target genes for the 12 dynamically changing miRNAs using Gene Ontology (GO) **(A)** and Kyoto Encyclopedia of Genes and Genomes (KEGG) **(B)** pathways.

**Figure 5:**
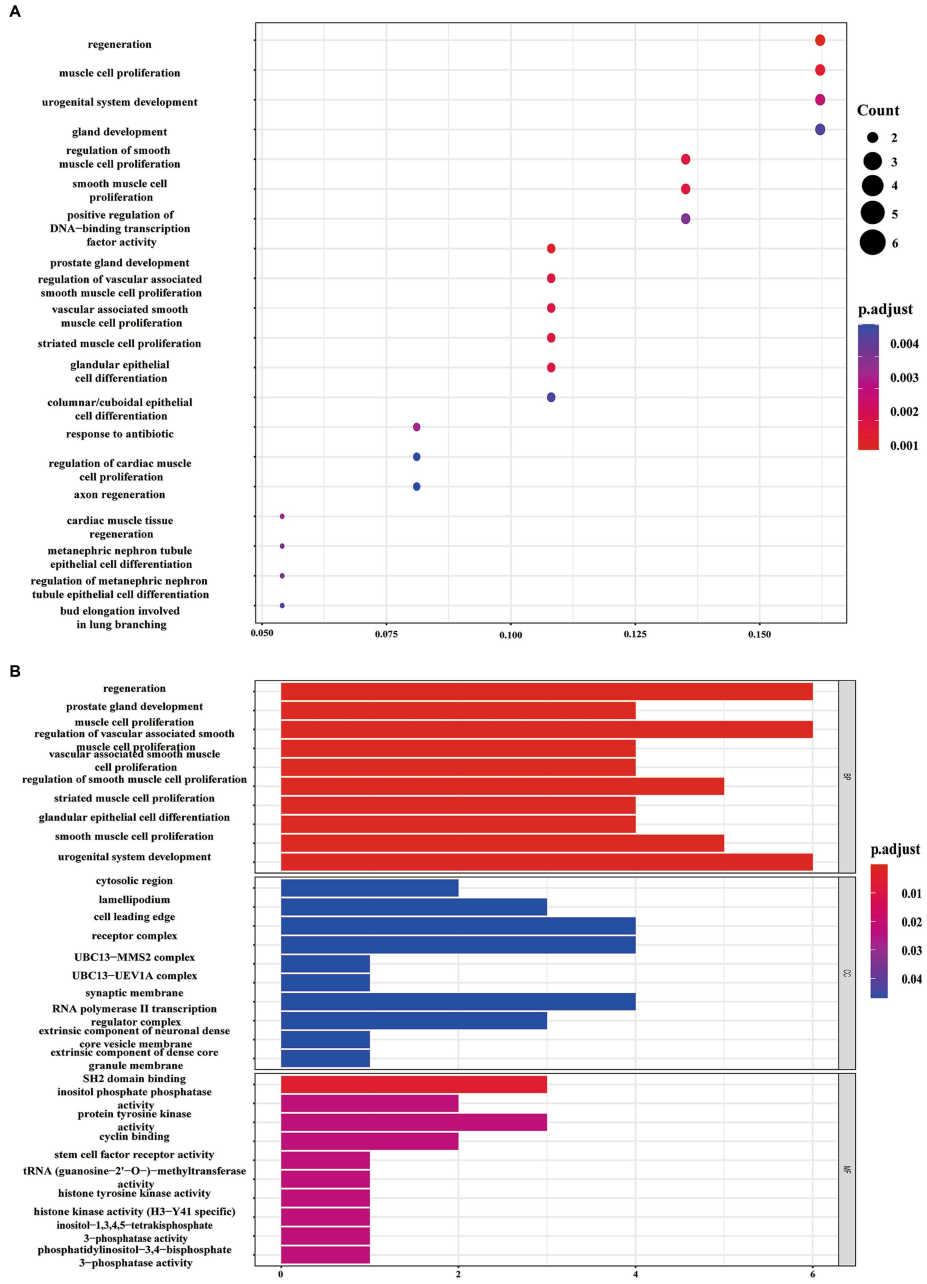
Enrichment analysis of target genes for the four uniquely regulated miRNAs by PCSK9i using Gene Ontology (GO) **(A)** and Kyoto Encyclopedia of Genes and Genomes (KEGG) **(B)** pathways.

Using information from TargetScan 8.0, miRTarBase, and insights from relevant studies (23, 31), we determined the target genes of 12 miRNAs and four miRNAs that might play a role in making AS plaques more stable due to PCSK9i treatment. The downstream target genes of these miRNAs were shown visually in Cytoscape (Figures 3A, B). Notably, the downstream gene prediction of mmu-miR-411-5p showed no intersection in either the TargetScan or miRTarBase databases; thus, it was excluded from further analysis. Further investigation of the target genes included Gene Ontology (GO) analysis which focused on key functions, such as cellular components (CC), biological processes (BP), and molecular functions (MF), as well as significant enrichment analysis in the Kyoto Encyclopedia of Genes and Genomes (KEGG) pathways. The results revealed that the target genes influenced by the 12 miRNAs were involved in various tissue development processes like neurogenesis and myogenesis. These genes were also associated with signaling pathways such as Wnt and TGF-β (Figures 4A,B). Moreover, the target genes of mmu-miR-186-5p, mmu-miR-222, mmu-miR-375-3p, and mmu-miR-494-3p clustered functionally in cardiovascular smooth muscle cell proliferation, migration, and regulation (Figures 5A,B). These findings suggest that PCSK9i promotes the stabilization of AS plaques by affecting these genes.

### 3.4 PCSK9i regulates the expression levels of Wipf2, Pdk1 and Yap1 by targeting mmu-miR-186-5p and mmu-miR-375-3p

**Figure 6:**
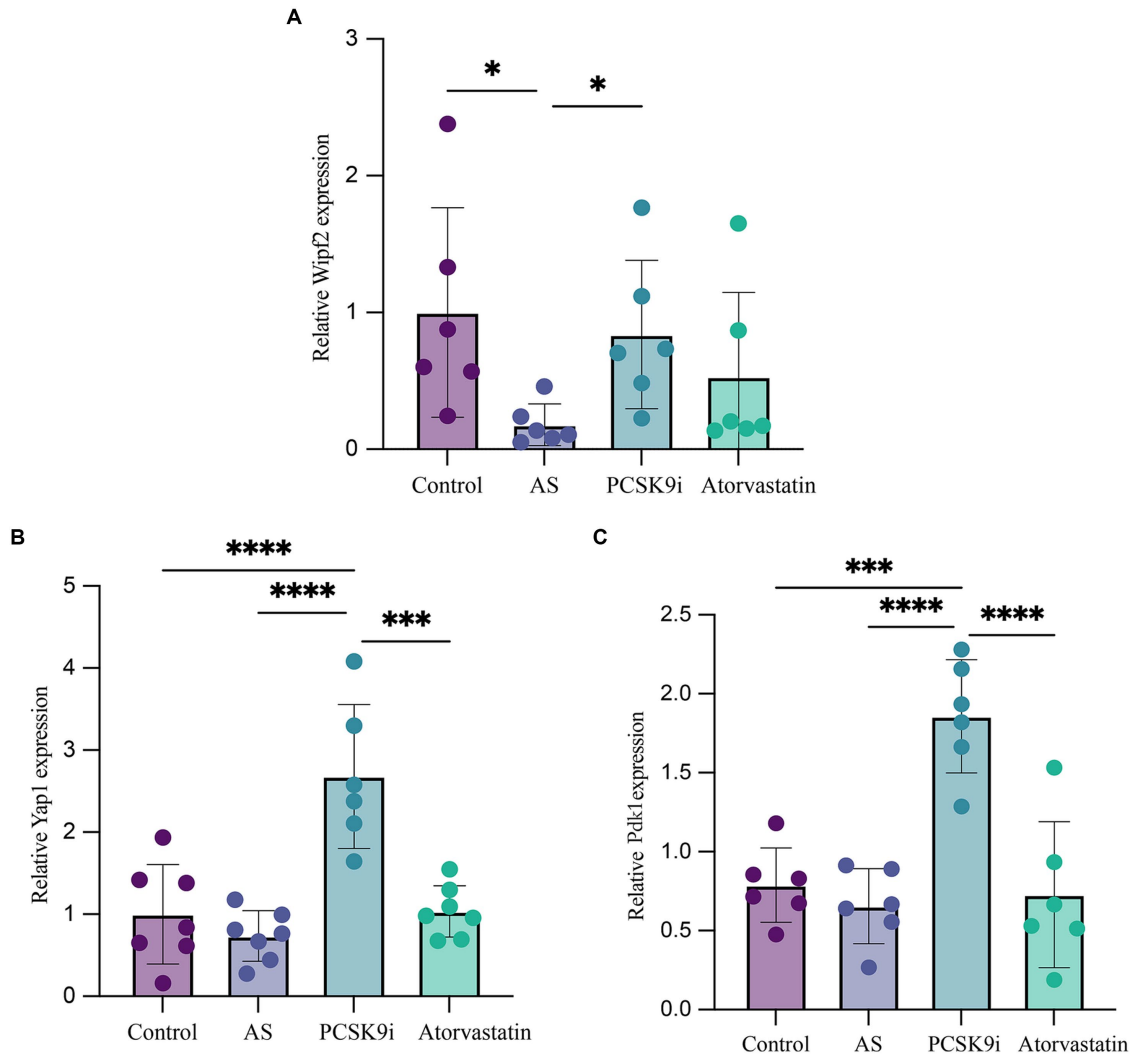
The expression of Wipf2, Pdk1 and Yap1 in aortic tissue of ApoE−/− mice in four groups was verified by RT-qPCR. **(A)** The relative mRNA level of Wipf2. **(B)** The relative mRNA level of Yap1. **(C)** The relative mRNA level of Pdk1. ***p < 0.001, **p < 0.01, and *p < 0.05.

We selected mmu-miR-186-5p and mmu-miR-375-3p for further investigation and employed bioinformatics methods to identify Wipf2, Pdk1, and Yap1 as downstream genes associated with both miRNAs in AS. To validate these findings, we extracted total RNA from the aortas of the four groups, followed by RT-qPCR. The results demonstrated that the expression of Wipf2 in the AS group was significantly lower than that in the control group (*p* < 0.05), while in the PCSK9i group, its expression was significantly upregulated (*p* < 0.05; Figure 6A). Notably, no significant differences in Pdk1 or Yap1 expression were observed between the control and AS groups. However, in the PCSK9i group, their expression was significantly increased (*p* < 0.05; Figures 6B,C). These findings suggest that Wipf2, Pdk1, and Yap1 are individually regulated by mmu-miR-186-5p and mmu-miR-375-3p under the influence of PCSK9i, and may play a role in promoting AS plaque stability.

## 4 Discussion

Atherosclerosis, a multifaceted disease, involves dysregulated lipid metabolism, vascular inflammation, and the participation of various cell types, including endothelial and smooth muscle cells. Its hallmark is the formation of AS plaques within the vascular lumen, leading to luminal narrowing and subsequent adverse consequences (36–38). The evolution of AS plaques comprises two phases: an initial stage characterized by intravascular lipid accumulation and foam cell formation in the vessel walls, resulting in unstable plaques, known as lesion progression; followed by a sequence of reparative processes aimed at preventing severe intravascular thrombus formation after prolonged inflammatory stimuli or plaque rupture. This healing phase, termed lesion healing, typically involves extensive proliferation of smooth muscle cells and their migration from the media to the intima (39). Research highlights the advantageous role of statin medications and other lipid-lowering therapies in promoting plaque healing (40, 41). A reduction in peripheral lipid levels is associated with significantly decreased expression of inflammatory markers and interstitial collagenase within atherosclerotic lesions, indicating a relationship between lipid reduction and plaque stability (42–45).

PCSK9 is a protein primarily expressed and secreted by hepatic tissues. Upon binding to the LDLR, PCSK9 forms a PCSK9-LDLR complex, preventing LDLR conformational changes and leading to the lysosomal degradation of LDLR-LDL complexes, thereby promoting LDLR degradation (46–48). PCSK9i reduced the formation of PCSK9-LDLR complexes, thereby increasing the number of LDLRs on the surface of hepatocytes. This enhances the uptake and degradation of circulating LDL by hepatocytes, resulting in reduced blood lipid levels and plaque stability (49). In this study, we observed a significant increase in circulating levels of TC, TG, and LDL-C, and a marked decrease in HDL-C levels in the AS group. After 8 weeks of drug intervention, both the PCSK9i and statin groups exhibited reductions in TC, TG, and LDL-C levels, along with an increase in HDL-C levels. Further tissue staining revealed a reduction in plaque volume and an improvement in lipid accumulation in the aortic roots of mice in both the PCSK9i and statin groups, with the PCSK9i group showing a more pronounced advantage (*p* < 0.05). These findings confirm that PCSK9i may enhance plaque stability by improving circulating lipid levels.

As a class of small non-coding RNAs consisting of approximately 18–22 nucleotides, miRNAs play a crucial role in various biological activities, such as cell proliferation, migration, differentiation, and regulation of cytokine production (50). In the pathological process of AS, studies have indicated that miRNAs are involved in regulating plaque progression and extracellular matrix remodeling (51–53). To further explore the specific mechanism by which PCSK9i stabilized plaques in AS lesions, we extracted total RNA from mouse aortic tissues and performed PCR ARRAY. The results revealed dynamic changes in 12 miRNAs, including mmu-miR-134, mmu-miR-141-5p, mmu-miR-17-3p, mmu-miR-195-3p, mmu-miR-210, mmu-miR-33–5p, mmu-miR-410, mmu-miR-411-5p, mmu-miR-499, mmu-miR-672-5p, mmu-miR-675-3p, and mmu-miR-301b, among the AS, PCSK9i, and statin groups. Through further functional prediction and pathway analysis using bioinformatics methods, we found that these differentially expressed miRNAs may be involved in regulating the generation of various tissues, such as neurones and muscles, and signal pathways such as Wnt and TGF-beta. Previous studies have also demonstrated the involvement of these miRNAs in the regulation of lipid accumulation, energy metabolism, production of vascular inflammatory factors, and proliferation, migration, and apoptosis of endothelial and smooth muscle cells (15–17, 19, 21, 54–58), indicating their close relevance to the development of AS. These findings highlight the crucial role of miRNAs in the pathological process of AS and suggest that statins and the PCSK9i may exert their anti-AS effects by modulating specific miRNAs.

In addition to the miRNAs mentioned above, PCSK9i was also found to significantly downregulate the expression levels of mmu-miR-186-5p, mmu-miR-222, mmu-miR-375-3p, and mmu-miR-494-3p. Previous studies by Naeli (59) and Desita (60) also suggested an association between PCSK9 protein and mmu-miR-222 and mmu-miR-494-3p. Research has indicated that miR-186-5p is significant upregulated in AS lesions, making it a potential novel biomarker for AS diagnosis (61–63). Functionally, miR-186-5p has been shown to promote vascular smooth muscle cell (VSMC) proliferation, migration, and phenotypic regulation (63, 64). Additionally, studies have revealed that miR-186-5p levels increase in exosomes released by macrophages stimulated with oxidized low-density lipoproteins (Ox-LDL), enhancing VSMC survival and invasiveness while suppressing apoptosis (65). These findings suggested that miR-186-5p participates in various mechanisms of AS progression, and similarly, miR-375-3p is involved in multiple biological processes in AS lesions. Research has indicated its significant upregulation in AS models and its inhibition leads to reduced intracellular lipid accumulation (66, 67). Conversely, miR-375-3p overexpression promotes endothelial cell proliferation, migration, and phenotypic transition, potentially promoting CAS development by targeting XIAP (68). Studies have shown that miR-375-3p inhibitors exert a protective effect against high glucose- or hypoxia-induced endothelial progenitor cell (EPC) injury, and enhance the antioxidant capacity in a myocardial fibroblast model (69, 70). Our GO and KEGG functional analyses of the downstream target genes of miR-186-5p and miR-375-3p in muscle cells further indicated their crucial roles in processes such as proliferation, migration, and regulation, which are closely related to plaque stability. These results suggest that PCSK9i facilitates the transition of atherosclerotic plaques to stable plaques by modulating the expression levels of mmu-miR-186-5p and mmu-miR-375-3p.

Furthermore, using TargetScan and miRTarBase, we identified Wipf2, PDK1, and YAP1 as downstream target genes of miR-186-5p and miR-375-3p, consistent with previous experimental results (23, 31, 71). Subsequently, RT-qPCR was performed to validate these findings. The results demonstrated a significant upregulation of Wipf2, Pdk1 and Yap1 expression in the PCSK9i group compared to the AS group. The Wipf2 (Wiskott-Aldrich Syndrome protein Family Member 2) has been implicated in various biological events, as it participates in the phagocytosis of Aspergillus fumigatus by human bronchial epithelial cells (72). Moreover, Wipf2 is involved in competing for endogenous RNA (ceRNA) modes and promotes tumor development in hepatocellular carcinoma (73). Additionally, the inhibition of Wipf2 expression enhances cellular toxicity and vaccine responsiveness (74). Tao et al. demonstrated that miR-186-5p influenced the proliferation and apoptosis of AS cells by targeting Wipf2 (23). As a protein kinase, Pdk1 is involved in multiple biological processes by phosphorylating and regulating the activity of other proteins (75). Studies have shown that Pdk1 is sensitive to oxidative stress, regulates autophagy through the Akt/mTOR pathway, and plays a critical role in platelet-derived growth factor-induced endothelial cell migration (76, 77). Previous studies have shown that the overexpression of miR-210 and miR-375-3p promotes AS development and increases plaque vulnerability by targeting Pdk1 to regulate endothelial cell apoptosis and promote VSMC phenotypic transition, whereas elevated Pdk1 levels increase the survival capacity of Ox-LDL-treated endothelial cells (31, 58). Additionally, we observed a significant upregulation of Yap1 in the PCSK9i group. As a transcriptional coactivator, Yap1 expression is significantly increased in stable plaques (78). Previous research has demonstrated that elevated Yap1 levels promote the adoption of a fibroblast-like phenotype (79, 80). Cheng et al. (81) also indicated the importance of the miR-375-3p/Yap1 axis in small-cell lung cancer cell proliferation, migration, and invasion. In our study, we speculated that PCSK9i may positively impact the transformation of unstable plaques into stable plaques by targeting the miR-186-5p/Wipf2 and miR-375-3p/Pdk1/Yap1 axes to regulate the VSMC phenotypic transition.

However, our study had some limitations that should be acknowledged. Although we identified the potential involvement of PCSK9i in the regulation of the miR-186-5p/Wipf2 and miR-375-3p/Pdk1/Yap1 axes to exert anti-AS effects, multiple miRNAs are known to regulate the expression of Wipf2, PDK1, and YAP1. Moreover, considering that VSMC migration and phenotypic transition are regulated by various pathways (82, 83), the detailed mechanisms underlying PCSK9i’s promotion of AS plaque stability require further exploration.

In conclusion, our findings confirm the crucial roles of mmu-miR-134, mmu-miR-141-5p, mmu-miR-17-3p, mmu-miR-195-3p, mmu-miR-210, mmu-miR-33–5p, mmu-miR-410, mmu-miR-411-5p, mmu-miR-499, mmu-miR-672-5p, mmu-miR-675-3p, and mmu-miR-301b in AS development. In addition, we elucidated the beneficial role of PCSK9i in the treatment of AS. Subcutaneous injection of PCSK9i appeared to exert anti-AS effects by targeting the miR-186-5p/Wipf2 and miR-375-3p/Pdk1/Yap1 axes, thereby promoting the transformation of AS plaques into more stable forms.

## Data availability statement

The original contributions presented in the study are included in the article/Supplementary material, further inquiries can be directed to the corresponding author.

## Ethics statement

The animal study was approved by Experimental Animal Ethics Committee, School of Basic Medicine, Jilin University. The study was conducted in accordance with the local legislation and institutional requirements.

## Author contributions

YZ: Writing – original draft. NL: Writing – original draft. JZ: Writing – review & editing. LZ: Writing – review & editing.

## Footnotes

^1^https://www.targetscan.org/

^2^https://mirtarbase.cuhk.edu.cn/

^3^https://david-d.ncifcrf.gov/

^4^https://pga.mgh.harvard.edu/primerbank/index.html
