# Corrigendum: PCSK9i promoting the transformation of AS plaques into a stable plaque by targeting the miR-186-5p/Wipf2 and miR-375-3p/Pdk1/Yap1 in ApoE–/– mice

**DOI:** 10.3389/fmed.2024.1416622

**Published:** 2024-06-04

**Authors:** Yanlong Zhao, Ning Liu, Jifeng Zhang, Lei Zhao

**Affiliations:** ^1^Department of Cardiology, The Second Hospital of Jilin University, Changchun, Jilin, China; ^2^School of Pharmaceutical Sciences, Jilin University, Changchun, Jilin, China

**Keywords:** atherosclerosis, PCSK9i, miRNA, atorvastatin, atherosclerotic plaque

In the published article, there was an error in the Funding statement. The funding statement for the Department of Finance of Jilin Province, China was displayed as “3D5204920429”. The correct statement is “Department of Finance of Jilin Province, China (No. 2020SCZT043).” The correct Funding statement appears below.

